# The Impact of Estimating High-Resolution Tropospheric Gradients on Multi-GNSS Precise Positioning

**DOI:** 10.3390/s17040756

**Published:** 2017-04-03

**Authors:** Feng Zhou, Xingxing Li, Weiwei Li, Wen Chen, Danan Dong, Jens Wickert, Harald Schuh

**Affiliations:** 1 Engineering Center of SHMEC for Space Information and GNSS, East China Normal University, No. 500 Dongchuan Road, Shanghai 200241, China; zhouforme@163.com (F.Z.); wchen@sist.ecnu.edu.cn (W.C.); dndong@cs.ecnu.edu.cn (D.D); 2 Shanghai Key Laboratory of Multidimensional Information Processing, East China Normal University, No. 500 Dongchuan Road, Shanghai 200241, China; 3 German Research Centre for Geosciences GFZ, Telegrafenberg, Potsdam 14473, Germany; lxlq109121@gmail.com (X.L.); wickert@gfz-potsdam.de (J.W.); schuh@gfz-potsdam.de (H.S.); 4 College of Surveying and Geo-informatics, Tongji University, Shanghai 200092, China; 5 Faculty VI Planning Building Environment, Technical University of Berlin, Berlin 10623, Germany

**Keywords:** GNSS, tropospheric gradients, precise point positioning (PPP), temporal resolution, elevation cutoff angle

## Abstract

Benefits from the modernized US Global Positioning System (GPS), the revitalized Russian GLObal NAvigation Satellite System (GLONASS), and the newly-developed Chinese BeiDou Navigation Satellite System (BDS) and European Galileo, multi-constellation Global Navigation Satellite System (GNSS) has emerged as a powerful tool not only in positioning, navigation, and timing (PNT), but also in remote sensing of the atmosphere and ionosphere. Both precise positioning and the derivation of atmospheric parameters can benefit from multi-GNSS observations. In this contribution, extensive evaluations are conducted with multi-GNSS datasets collected from 134 globally-distributed ground stations of the International GNSS Service (IGS) Multi-GNSS Experiment (MGEX) network in July 2016. The datasets are processed in six different constellation combinations, i.e., GPS-, GLONASS-, BDS-only, GPS + GLONASS, GPS + BDS, and GPS + GLONASS + BDS + Galileo precise point positioning (PPP). Tropospheric gradients are estimated with eight different temporal resolutions, from 1 h to 24 h, to investigate the impact of estimating high-resolution gradients on position estimates. The standard deviation (STD) is used as an indicator of positioning repeatability. The results show that estimating tropospheric gradients with high temporal resolution can achieve better positioning performance than the traditional strategy in which tropospheric gradients are estimated on a daily basis. Moreover, the impact of estimating tropospheric gradients with different temporal resolutions at various elevation cutoff angles (from 3° to 20°) is investigated. It can be observed that with increasing elevation cutoff angles, the improvement in positioning repeatability is decreased.

## 1. Introduction

Electromagnetic propagation delays due to tropospheric horizontal gradients can affect high-precision radiometric space geodetic techniques results such as Very Long Baseline Interferometry (VLBI), Global Navigation Satellite Systems (GNSS), Doppler Orbitography and Radiopositioning Integrated by Satellite (DORIS) [1,2,3,4,5]. Actually, tropospheric gradients can be significant when observations at low elevation angles are used. However, the use of low elevation ($3^{\circ }\leq e\leq 15^{\circ }$) observations is essential to improve the accuracy of GNSS analysis, in particular to decorrelate the estimated station heights and the tropospheric zenith total delays (ZTDs). In the simulation of MacMillan [2], it was found that, in the case of VLBI observations, a gradient anomaly of 1 mm could produce a range error of about 65 mm at 7° elevation and 33 mm at 10° elevation, respectively. Boehm and Schuh [6] indicated that a gradient anomaly of 0.1 mm was equivalent to a range error of about 10 mm at 5° elevation and a horizontal station position error of about 1 mm, as well. Iwabuchi et al. [7] pointed out that estimating the tropospheric gradients could also improve the accuracy of the tropospheric ZTD estimates. The simulated and practical results confirmed that a north–south horizontal gradient anomaly of 1 mm gave rise to about 1 mm negative ZTD biases.

To consider tropospheric gradients, Herring [8] proposed a tropospheric gradient model based on a “tilted” atmospheric assumption for VLBI analysis and showed that the accuracy of the baseline length estimates could be improved. MacMillan [2] also confirmed that VLBI baseline repeatability can be improved by up to 8 mm with a simple gradient model. A more accurate gradient model, especially at low elevation angles, was proposed in Chen and Herring [3]. In addition to VLBI, tropospheric gradients were also considered in Global Positioning System (GPS) analysis. Bar-Sever et al. [4] first implemented MacMillan’s gradient model into GPS data analysis. By using the GPS precise point positioning (PPP) technique [9], the results demonstrated that the gradient model improved the station position repeatability in most cases. By using PPP and network processing, improvements in the precision of station position estimates were also demonstrated in other previous studies [10]. The estimation of tropospheric horizontal gradients together with zenith delays is now commonly carried out by a wide range of GNSS processing software, such as US GPS Analysis at Massachusetts Institute of Technology (GAMIT) and Swiss Bernese GNSS software. The piecewise gradient parameters are usually estimated on a daily basis to avoid large variations and jumps in the gradients and to reduce the number of parameters [11].

The previous studies of tropospheric gradients are mainly in VLBI and GPS data analysis. Currently, the world of satellite navigation and positioning is entering a new era of multi-GNSS as the GPS and the Russian GLObal NAvigation Satellite System (GLONASS) are being modernized and new constellations, like the Chinese BeiDou Navigation Satellite System (BDS) and the European Galileo Navigation Satellite System (Galileo), are being developed. The new navigation satellite systems can act either as a supplement to the currently used systems, like GPS and GLONASS, or as a stand-alone system in some regions [12,13,14]. With more and more satellites being in view, multi-GNSS precise positioning has become a very hot research topic [15,16,17]. Li et al. [18] indicated that the tropospheric gradients, derived from multi-GNSS, agree slightly better with the European Centre for Medium-Range Weather Forecasts (ECMWF) derived gradients than the GPS-only derived gradients. Since the gradients from the GNSS techniques are averaged over a certain period, the gradients with lower temporal resolutions will underestimate the gradient magnitude. Lu et al. [19] first demonstrated the benefits of multi-GNSS processing for the retrieval of high-resolution tropospheric gradients, as well as for the improvement of precise positioning. 

In this study, observation data from 134 globally-distributed stations from the International GNSS Service (IGS) Multi-GNSS Experiment (MGEX) [20] network is selected. The datasets are processed by estimating tropospheric gradients with eight different temporal resolutions, from 1 h to 24 h, to investigate the impact of estimating high-resolution gradients on position estimates. In addition to the impact of temporal resolution, the impact of different constellation combinations, i.e., GPS-, GLONASS-, BDS-only, GPS + GLONASS, GPS + BDS and GPS + GLONASS + BDS + Galileo is investigated, as well as different elevation cutoff angles from 3° to 20°. The remaining paper is organized as follows: Section 2 presents the ionosphere-free (IF) observation model of multi-GNSS PPP. After a brief statement about the data and processing strategy in Section 3, the impact of estimating high-resolution tropospheric gradients on station position estimates is discussed in detail in Section 4. Finally, conclusions are drawn in Section 5.

## 2. Multi-GNSS Ionosphere-Free PPP Observation Model

The undifferenced GNSS ionosphere-free observations for pseudorange *P* and carrier phase *L* can be expressed as follows:

(1) $$P_{r,IF}^{s}=\rho_{r}^{s}+t_{r}-t^{s}+T_{r}^{s}+\varepsilon_{r,IF}^{s}$$

(2) $$L_{r,IF}^{s}=\rho_{r}^{s}+t_{r}-t^{s}+T_{r}^{s}+N_{r,IF}^{s}+\xi_{r,IF}^{s}$$

where indices s and r refer to the satellite and receiver, respectively; $\rho_{r}^{s}$ denotes the geometric distance between the satellite and receiver; $t_{r}$ and $t^{s}$ are the clock offsets of the receiver and satellite; $T_{r}^{s}$ is the slant tropospheric delay; $N_{r,IF}^{s}$ is the ionosphere-free phase ambiguity; $\varepsilon_{r,IF}^{s}$ and $\xi_{r,IF}^{s}$ are the sum of measurement noise and multipath error for the ionosphere-free pseudorange and carrier phase observations. Note that all of the variables in Equations (1) and (2) are expressed in meters.

Normally, the slant tropospheric delay $T_{r}^{s}$ is modeled by the sum of hydrostatic, wet and gradient delays [18] as follows:

(3) $$T_{r}^{s}=mf_{h}(e)\cdot Z_{h}+mf_{w}(e)\cdot Z_{w}+mf_{g}(e)\cdot [G_{ns}\cdot \cos(a)+G_{ew}\cdot \sin(a)]$$

where a and e is the azimuth and elevation angle of the satellite, respectively. $Z_{h}$ denotes zenith hydrostatic delay (ZHD), which can be modeled accurately using empirical models, such as Saastamoinen [21]. $mf_{h}(e)$ and $mf_{w}(e)$ are the hydrostatic and wet mapping functions that can be retrieved with Global Mapping Function (GMF) [22]. $mf_{g}(e)$ is the gradient mapping function [3]. In the GNSS based troposphere modeling, the zenith wet delay (ZWD) $Z_{w}$ and the gradient vector $G={\left(\begin{array}{cc} G_{ns} & G_{ew} \end{array}\right)}^{T}$ with north–south and east–west components, are usually estimated as unknowns along with other parameters in PPP processing.

Substituting Equation (3) into Equations (1) and (2), and applying the IGS MGEX precise satellite orbit and clock products, the linearized observation model within the quad-constellation (GPS + GLONASS + BDS + Galileo) context can be rewritten as:

(4) $$\left\{ \begin{array}{ll} p_{r,IF}^{G}= & u_{r}^{G}\cdot x+t_{r}+mf_{w}(e)\cdot Z_{w}+mf_{g}(e)\cdot \cos(a)\cdot G_{ns}\\ & +mf_{g}(e)\cdot \sin(a)\cdot G_{ew}+\varepsilon_{r,IF}^{G}\\ p_{r,IF}^{R}= & u_{r}^{R}\cdot x+t_{r}+IFB_{R_{k},G}+mf_{w}(e)\cdot Z_{w}+mf_{g}(e)\cdot \cos(a)\cdot G_{ns}\\ & +mf_{g}(e)\cdot \sin(a)\cdot G_{ew}+\varepsilon_{r,IF}^{R}\\ p_{r,IF}^{C}= & u_{r}^{C}\cdot x+t_{r}+ISB_{C,G}+mf_{w}(e)\cdot Z_{w}+mf_{g}(e)\cdot \cos(a)\cdot G_{ns}\\ & +mf_{g}(e)\cdot \sin(a)\cdot G_{ew}+\varepsilon_{r,IF}^{C}\\ p_{r,IF}^{E}= & u_{r}^{E}\cdot x+t_{r}+ISB_{E,G}+mf_{w}(e)\cdot Z_{w}+mf_{g}(e)\cdot \cos(a)\cdot G_{ns}\\ & +mf_{g}(e)\cdot \sin(a)\cdot G_{ew}+\varepsilon_{r,IF}^{E} \end{array}\right.$$

(5) $$\left\{ \begin{array}{ll} l_{r,IF}^{G}= & u_{r}^{G}\cdot x+t_{r}+mf_{w}(e)\cdot Z_{w}+mf_{g}(e)\cdot \cos(a)\cdot G_{ns}\\ & +mf_{g}(e)\cdot \sin(a)\cdot G_{ew}+N_{r,IF}^{G}+\varepsilon_{r,IF}^{G}\\ l_{r,IF}^{R}= & u_{r}^{R}\cdot x+t_{r}+mf_{w}(e)\cdot Z_{w}+mf_{g}(e)\cdot \cos(a)\cdot G_{ns}\\ & +mf_{g}(e)\cdot \sin(a)\cdot G_{ew}+N_{r,IF}^{R}+\varepsilon_{r,IF}^{R}\\ l_{r,IF}^{C}= & u_{r}^{C}\cdot x+t_{r}+mf_{w}(e)\cdot Z_{w}+mf_{g}(e)\cdot \cos(a)\cdot G_{ns}\\ & +mf_{g}(e)\cdot \sin(a)\cdot G_{ew}+N_{r,IF}^{C}+\varepsilon_{r,IF}^{C}\\ l_{r,IF}^{E}= & u_{r}^{E}\cdot x+t_{r}+mf_{w}(e)\cdot Z_{w}+mf_{g}(e)\cdot \cos(a)\cdot G_{ns}\\ & +mf_{g}(e)\cdot \sin(a)\cdot G_{ew}+N_{r,IF}^{E}+\varepsilon_{r,IF}^{E} \end{array}\right.$$

where the indices *G*, *R*, *C* and *E* refer to GPS, GLONASS, BDS and Galileo, respectively; $p_{r,IF}^{s}$ and $l_{r,IF}^{s}$ denote observed minus computed (OMC) values of the pseudorange and carrier phase observables for the satellite system s (s=G, R, C, or E); $u_{r}^{s}$ is the unit vector of the component from the receiver to the satellite; x is the vector of the receiver position increments relative to a priori position; $R_{k}$ denotes GLONASS satellite with frequency factor k, which is used for the computation of the carrier phase frequencies of the individual GLONASS satellites. It is noted that one ISB parameter is introduced for the BDS or Galileo satellites; while, the $IFB_{R_{k},G}$ parameter for each station and GLONASS satellite pair in this study is actually the combination of original inter-system bias (ISB) of GPS and GLONASS as well as inter-frequency code bias of GLONASS. S is the estimates vector:

(6) $$S={\left[x,t_{r},IFB_{R_{k},G},ISB_{C,G},ISB_{E,G},Z_{w},G_{ns},G_{ew},N_{r,IF}\right]}^{T}$$

## 3. Experimental Data and Processing Strategy

### 3.1. Dataset

**Figure 1:**
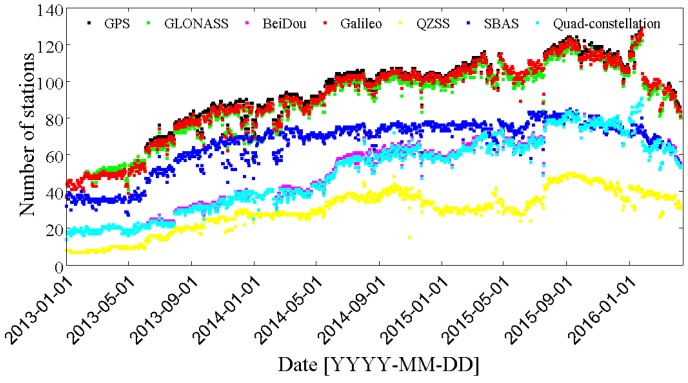
Number of MGEX ground tracking stations.

**Figure 2:**
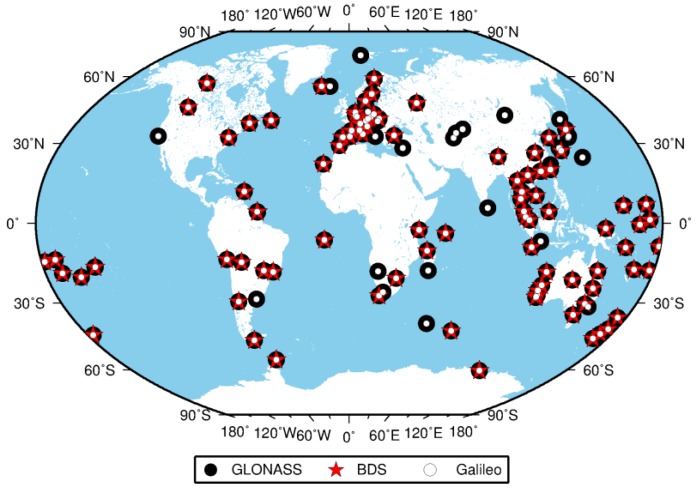
Geographical distribution of MGEX tracking stations and their supported navigation satellite constellations. Only GLONASS, BDS and Galileo are displayed, while GPS can be tracked by each station.

The MGEX network, setup by IGS, is designed to track, collect and analyze all available GNSS signals [23,24], including signals from BDS, Galileo, the Japanese Quasi-Zenith Satellite System (QZSS) and the Indian Regional Navigation Satellite System (IRNSS), as well as from the modernized GPS, GLONASS and space-based augmentation system (SBAS). The MGEX network has grown to more than 140 stations now supporting at least one of the new navigation systems (BDS, Galileo and QZSS) in addition to the legacy GPS, GLONASS and SBAS since 2011. Currently, about 120 stations are capable of tracking GLONASS and Galileo, and BDS is supported by more than 100 stations. The number of MGEX stations increased rapidly in recent years, as shown in Figure 1. The surging growth of quad-constellation (GPS + GLONASS + BDS + Galileo) receivers used within the MGEX network is expected to profit from the fast development of BDS and Galileo systems. Figure 2 displays the geographical distribution of 134 MGEX stations used in this study and also their supported constellations, except GPS, which can be tracked by all stations.

### 3.2. Processing Strategy

The Chinese Positioning and Navigation Data Analyst (PANDA) software [25] is used for the data processing in this study. Observations of a 31 day period in July 2016 from the selected 134 globally-distributed stations of the MGEX network are processed. Table 1 summarizes the detailed processing strategy for multi-GNSS PPP. Precise orbit and clock products at intervals of 5 min and 30 s, respectively, provided by MGEX (e.g., GFZ) [26] are employed. It is worth mentioning that, since 16 July 2014 the BDS Inclined Geo-Synchronous Orbit (IGSO) and Medium Earth Orbit (MEO) satellite antenna phase center offsets (PCOs) and variations (PCVs) estimated by Dilssner et al. [27] have been adopted for GFZ multi-GNSS processing. To be consistent, the same values are applied in our processing. Since the receiver PCOs/PCVs for BDS and Galileo are not provided by IGS, the same values for GPS are applied. The station coordinates from IGS weekly SINEX (Solution INdependent EXchange format) solutions are used as the ground truth for comparison and validation. However, precise coordinates of some stations are still not available. The daily coordinates of these stations are estimated using IGS GPS final orbit and clock products with the PANDA software in static mode. The averaged values from seven consecutive daily solutions are further computed as the truth. The datasets are processed in six different constellation combinations, i.e., GPS-only (G), GLONASS-only (R), BDS-only (C), GPS + GLONASS (GR), GPS + BDS (GC) and GPS + GLONASS + BDS + Galileo (GRCE) PPP. It is noted that Galileo-only PPP are currently hampered by the small number of usable satellites in view, while BDS-only PPP is restricted in the Asia–Pacific region [14]. One-hundred-thirty-four stations for GPS-, GLONASS-only and GPS + GLONASS, 61 stations for GPS + BDS and GPS + GLONASS + BDS + Galileo, and 48 stations for BDS-only are selected for analysis.

## 4. Results and Analysis

We define eight different solutions in terms of position repeatability, corresponding to estimating tropospheric gradients with eight different temporal resolutions. To simplify the subsequent description, these solutions are defined as “xxh_SYS”, where “xxh” represent estimating intervals, which can be 24, 12, 8, 6, 4, 3, 2 or 1 h. “SYS” is the combination of satellite systems, which can be G, R, C, GR, GC or GRCE. Specifically, “01h_G” represents GPS-only PPP solutions by estimating tropospheric gradients with a 1 h interval. Meanwhile, we also define a special case “No_SYS”, for PPP solutions without estimating tropospheric gradients. Specifically, “No_G” represents GPS-only PPP solutions without estimating tropospheric gradients.

### 4.1. Temporal Resolution Dependence

An elevation cutoff angle of 7° is adopted for the data processing in this part. In the following, the position repeatability derived from “No_SYS” solutions (as a reference) and “xxh_SYS” solutions are compared. The smaller ones represent improvements in the position repeatability.

**Figure 3:**
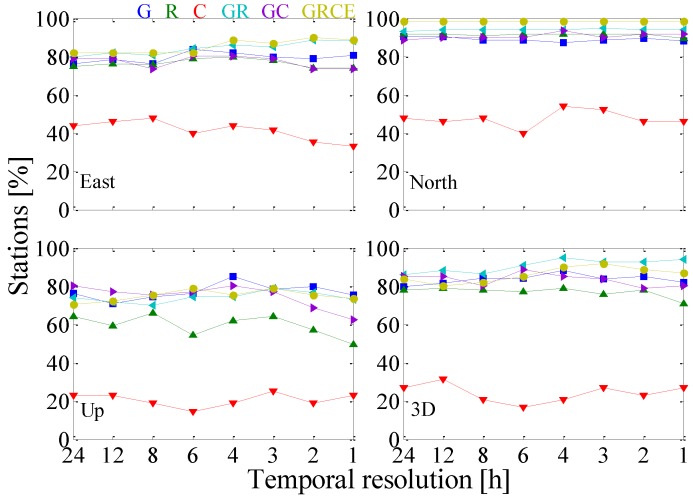
Station percentage with improved position repeatability (east, north, up and 3D components) derived from GPS-, GLONASS-, BDS-only, GPS + GLONASS, GPS + BDS and GPS + GLONASS + BDS + Galileo PPP solutions as a function of temporal resolutions with respect to “No_SYS” solutions. Different constellation combinations of G, R, C, GR, GC and GRCE are depicted in different colors.

Figure 3 displays station percentage with improved position repeatability in east, north, up and 3D components. For the east component, the percentage of improved stations for GPS + GLONASS and GPS + GLONASS + BDS + Galileo shows slightly linear growth with the varying temporal resolutions. The station percentage for GPS-, GLONASS-only and GPS + BDS decreased slightly when the temporal resolution is higher than 6 h. With estimating tropospheric gradients, the percentage of improved stations for BDS-only is less than 50%. For the north component, the station percentage of improved repeatability is independent of the varying temporal resolutions except that of BDS-only. It is interesting to note that more stations (more than 85% except BDS-only) are improved in the north component with estimating gradients than that in the other two components, indicating that the tropospheric gradients are probably more correlated with the north component. For the up component, estimating tropospheric gradients with the higher temporal resolutions of 1 h and 2 h reduces the percentage of stations dramatically, compared with the lower temporal resolutions, i.e., 24 h. From the 3D component in Figure 3, it can be observed that estimating tropospheric gradients has a negative impact on BDS-only PPP. Less than 40% of stations are improved in repeatability. Hence, we conclude that more than 60% of stations obtain larger positioning repeatability with estimating tropospheric gradients. This might be attributable to worse observation geometry, the signal instability, and the lower precision of the correction models for BDS (e.g., the PCO and PCV models).

**Figure 4:**
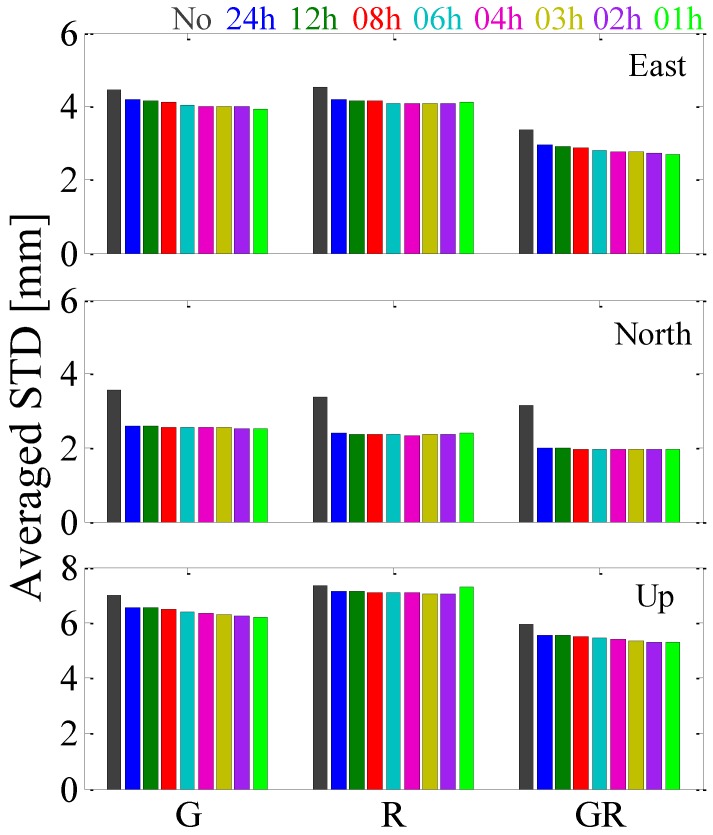
The averaged positioning repeatability of the selected 134 stations for GPS-, GLONASS-only and GPS + GLONASS PPP solutions.

Figure 4 illustrates the averaged positioning repeatability of the 134 stations for GPS-, GLONASS-only and GPS + GLONASS PPP. For the east component, compared with “No_G” solutions, the positioning repeatability of “24h_G” solutions is improved by 5.8%, from 4.45 to 4.19 mm. Compared with “24h_G” solutions, the positioning repeatability of “01h_G” solutions is further improved by 6.9%, from 4.19 to 3.90 mm, indicating that the position repeatability of GPS-only PPP solutions in the east component can be improved more by estimating tropospheric gradients with high temporal resolution. Compared with GPS-only PPP solutions, the improved percentage of positioning repeatability for GLONASS-only PPP solutions is small. Compared with the “24h_R” solutions, the positioning repeatability of “04h_R” solutions is only further improved by 2.6%, from 4.17 to 4.06 mm. For GPS + GLONASS PPP, compared with the “No_GR” solutions, the positioning repeatability of “24h_GR” solutions is improved by 12.8%, from 3.36 to 2.93 mm. Compared with the “24h_GR” solutions, the positioning repeatability of “01h_GR” solutions is further improved by 8.2%, from 2.93 to 2.69 mm. For the north component, from “No_SYS” to “24h_SYS” solutions the improved percentage is larger than the other two components for GPS-, GLONASS-only and GPS + GLONASS PPP. However, it can be observed that estimating tropospheric gradients with high temporal resolution has a small impact on PPP position estimates in the north component. For the up component, the performance is similar to that in the east component. It is obvious that estimating gradients with a 1 h interval is negative for GLONASS-only PPP.

**Figure 5:**
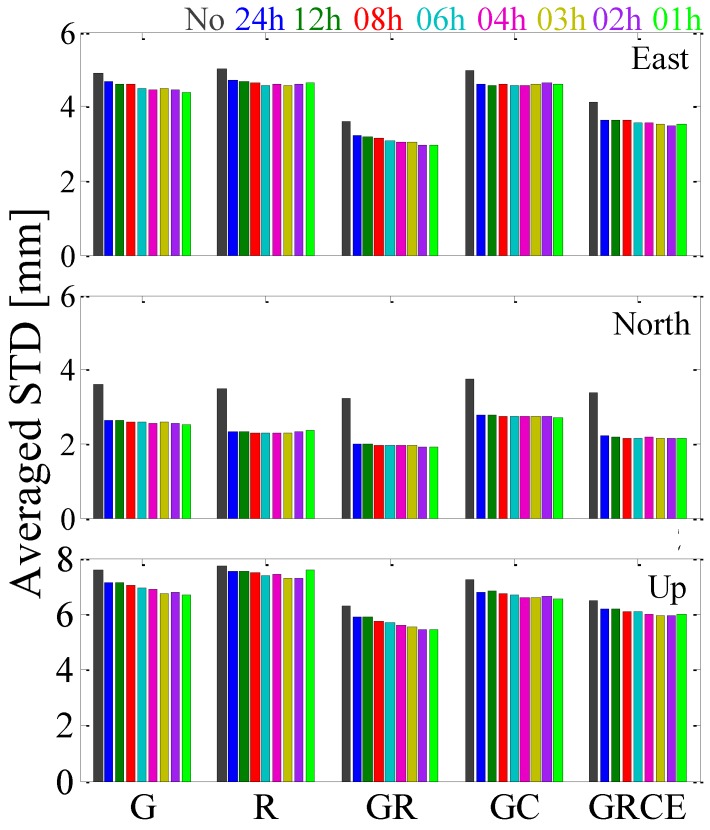
The averaged positioning repeatability of the selected 61 stations for GPS-, GLONASS-only, GPS + GLONASS, GPS + BDS and GPS + GLONASS + BDS + Galileo PPP solutions.

Figure 5 indicates the averaged positioning repeatability of the 61 stations for GPS-, GLONASS-only, GPS + GLONASS, GPS + BDS and GPS + GLONASS + BDS + Galileo PPP. In general, it can be observed that estimating tropospheric gradients with high temporal resolution has a very small impact on GPS + BDS PPP solutions and GPS + GLONASS + BDS + Galileo PPP position estimates in the east and north components. While, for the up component, compared with “No_GRCE” solutions, the positioning repeatability of “24h_GRCE” solutions is improved by 4.6%, from 6.50 to 6.20 mm. Compared with “24h_GRCE” solutions, the positioning repeatability of “02h_GRCE” solutions is further improved by 3.6%, from 6.20 to 5.98 mm.

### 4.2. Elevation Cutoff Angle Dependence

**Figure 6:**
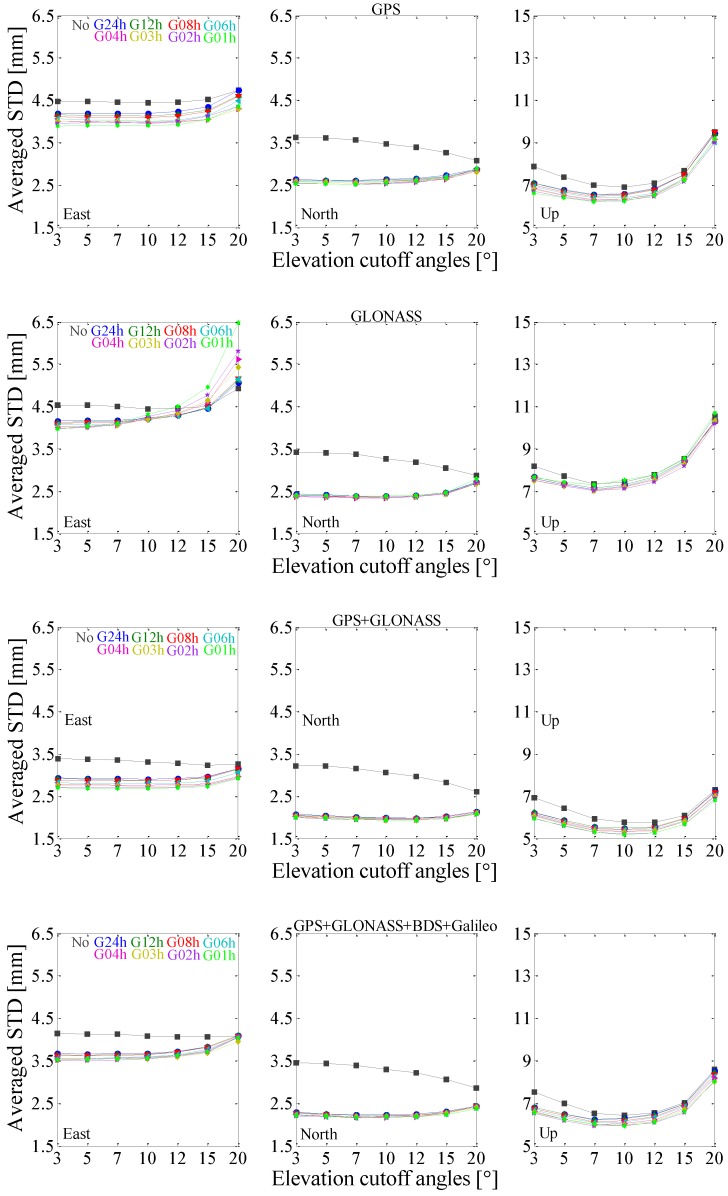
The averaged position repeatability as a function of satellite elevation cutoff angles of the 61 stations for GPS-, GLONASS-only, GPS + GLONASS and GPS + GLONASS + BDS + Galileo PPP solutions.

The impact of estimating tropospheric gradients with different temporal resolutions at various elevation cutoff angles is further investigated. Seven different elevation cutoff angles (3°, 5°, 7°, 10°, 12°, 15° and 20°) are considered. The averaged repeatability of the selected 61 stations for GPS-, GLONASS-only, GPS + GLONASS and GPS + GLONASS +BDS +Galileo PPP solutions are analyzed as shown in Figure 6. Generally, without estimating tropospheric gradients, the averaged repeatability of GPS-, GLONASS-only, GPS + GLONASS and GPS + GLONASS + BDS + Galileo PPP solutions in east component follows a decreased trend when the cutoff angles increase from 3° to 15°, while following an increased trend when the cutoff angles increase from 15° to 20°. For the north component, the averaged repeatability of PPP solutions follows a decreased trend with the cutoff angles increased. For the up component, the averaged repeatability of PPP solutions follows a decreased trend when the cutoff angles increase from 3° to 10°, while following an increased trend when the cutoff angles increase from 10° to 20°. While considering tropospheric gradients, for the east and north components, the averaged repeatability changes slowly when the cutoff angles increase from 3° to 10°, but increases more rapidly for the cutoff angles larger than 10°. For the up component, the averaged repeatability follows a decreased trend when the cutoff angles increase from 3° to 7°, while following an increased trend when the cutoff angles increase from 7° to 20°. It is apparent that with estimating tropospheric gradients the averaged repeatability is reduced only when the elevation cutoff angles are below 15°. The lower the elevation cutoff angles are, the more improvements in averaged repeatability can be observed compared to solutions without estimating gradients. The highest temporal resolution of 1 h shows its advantage on repeatability reduction over other resolutions for GPS-only, GPS + GLONASS and GPS + GLONASS + BDS + Galileo PPP in the case of any elevation cutoff angle. However, temporal resolution of 1 h shows a negative impact on GLONASS-only PPP position estimates. It is possible that 1 h resolution brings more estimated parameters, however, less GLONASS than GPS observations are recorded in most areas.

## 5. Conclusions

In this study, the impact of estimating tropospheric gradients with high temporal resolution on position estimates is extensively investigated with six different constellation combinations, i.e., GPS-, GLONASS-, BDS-only, GPS + GLONASS, GPS + BDS and GPS + GLONASS + BDS + Galileo PPP. Tropospheric gradients were estimated with eight different temporal resolutions, from 1 h to 24 h, to investigate various effects on station position repeatability. In addition, six different elevation cutoff angles, from 3° to 20°, were adopted to investigate their impact on position estimates.

First, the performance with estimating tropospheric gradients in different temporal resolutions was evaluated at a commonly used elevation cutoff angle of 7°. It is interesting that the station percentage of improved repeatability in the north component is independent of the temporal resolution, while it changes as a function of temporal resolution in the east and up components. However, more stations (more than 85%) are improved in the north component with estimated gradients than the other two components, indicating that the tropospheric gradients are probably more correlated with the north component. For currently BDS-only PPP, no tropospheric gradient estimation is a better choice. Compared with the solution by estimating gradients with low temporal resolution (i.e., 24 h), the averaged positioning repeatability can be further improved by 6.9% for GPS-only PPP, by 2.6% for GLONASS-only PPP, by 8.2% for GPS + GLONASS PPP and by 3.6% for GPS + GLONASS + BDS + Galileo PPP in the east component by using high-resolution gradients (i.e., 1 h or 2 h). For the up component, the performance is similar as that in the east component. Not surprisingly, the averaged positioning repeatability in the north component is independent on the temporal resolution.

The impact of estimating tropospheric gradients with different temporal resolutions at various elevation cutoff angles was also studied. The results show that with the increased elevation cutoff angles, the improvement in positioning repeatability is decreased. It was observed that, with estimating gradients, the averaged repeatability was reduced only when the elevation cutoff angles were below 15°. We found that the best positioning performance can be achieved at elevation cutoff angles of 7° or 10°.

## Figures and Tables

**Table 1:** Summary of multi-GNSS PPP processing strategies.

Items	Descriptions
Number of stations	134
Number of satellites	GPS: 32; GLONASS: 24; BDS: 14; Galileo: 10
Procedure	Integrated processing, all the observations from different GNSSs in one common parameter adjustment procedure
Estimator	Least squares (LSQ) estimator in batch mode
Observables	Undifferenced ionosphere-free combined observables from raw code and phase observations
Signal selection	GPS: L1/L2; GLONASS: L1/L2; BDS: B1/B2; Galileo: E1/E5a
Sampling rate	30 s
Elevation cutoff	3°/5°/7°/10°/12°/15°/20°
Observation weighting	A priori precision 0.6 m and 0.01 cycle for raw code and phase observations, respectively Elevation-dependent, 1 for $e>30^{\circ }$, otherwise 2×sin(e) [28]
Phase wind-up	Corrected [29]
Tropospheric delay	ZHD: corrected with global pressure and temperature (GPT) [30] model using the formulas of Saastamoinen [21] ZWD: estimated as a continuous piece-wise linear function (2 h parameter spacing), GMF [22] mapping function
Tropospheric gradients	Estimated as a continuous piece-wise linear function with different temporal resolutions
Tidal displacements	Solid Earth tide, pole tide, ocean tide loading corrections according to IERS Conventions 2010 [31]
Relativistic effect	Applied [32]
Sagnac effect	Applied [33]
Satellite antenna PCOs and PCVs	GPS and GLONASS: fixed to the values from igs08.atx [34]; BDS: fixed to nominal values (0.6, 0.0, 1.1 m) for GEO, and fixed to the estimated values provided by Dilssner et al. [27] for IGSO and MEO; Galileo: fixed to nominal values (0.2, 0.0, 0.6 m)
Receiver antenna PCOs and PCVs	PCO and PCV corrections for GPS and GLONASS are from igs08.atx; Corrections for BDS and Galileo are assumed the same with GPS
Receiver clock	Estimated as white noise
ISBs/IFBs	Estimated as daily constants without a priori constraints
Station coordinates	Estimated as static
Phase ambiguities	Estimated, constant for each continuous arc; float value
